# Impacts of the COVID-19 Pandemic on Mental Health and Potential Solutions in Different Members in an Ordinary Family Unit

**DOI:** 10.3389/fpsyt.2021.735653

**Published:** 2022-01-11

**Authors:** Limin Wang, Ghulam Nabi, Lirong Zuo, Yuefeng Wu, Dongming Li

**Affiliations:** Ministry of Education Key Laboratory of Molecular and Cellular Biology, Key Laboratory of Animal Physiology, Biochemistry and Molecular Biology of Hebei Province, College of Life Sciences, Hebei Normal University, Shijiazhuang, China

**Keywords:** COVID-19, ordinary family members, mental health disorders, crisis intervention measures, family unit

**Graphical Abstract:**
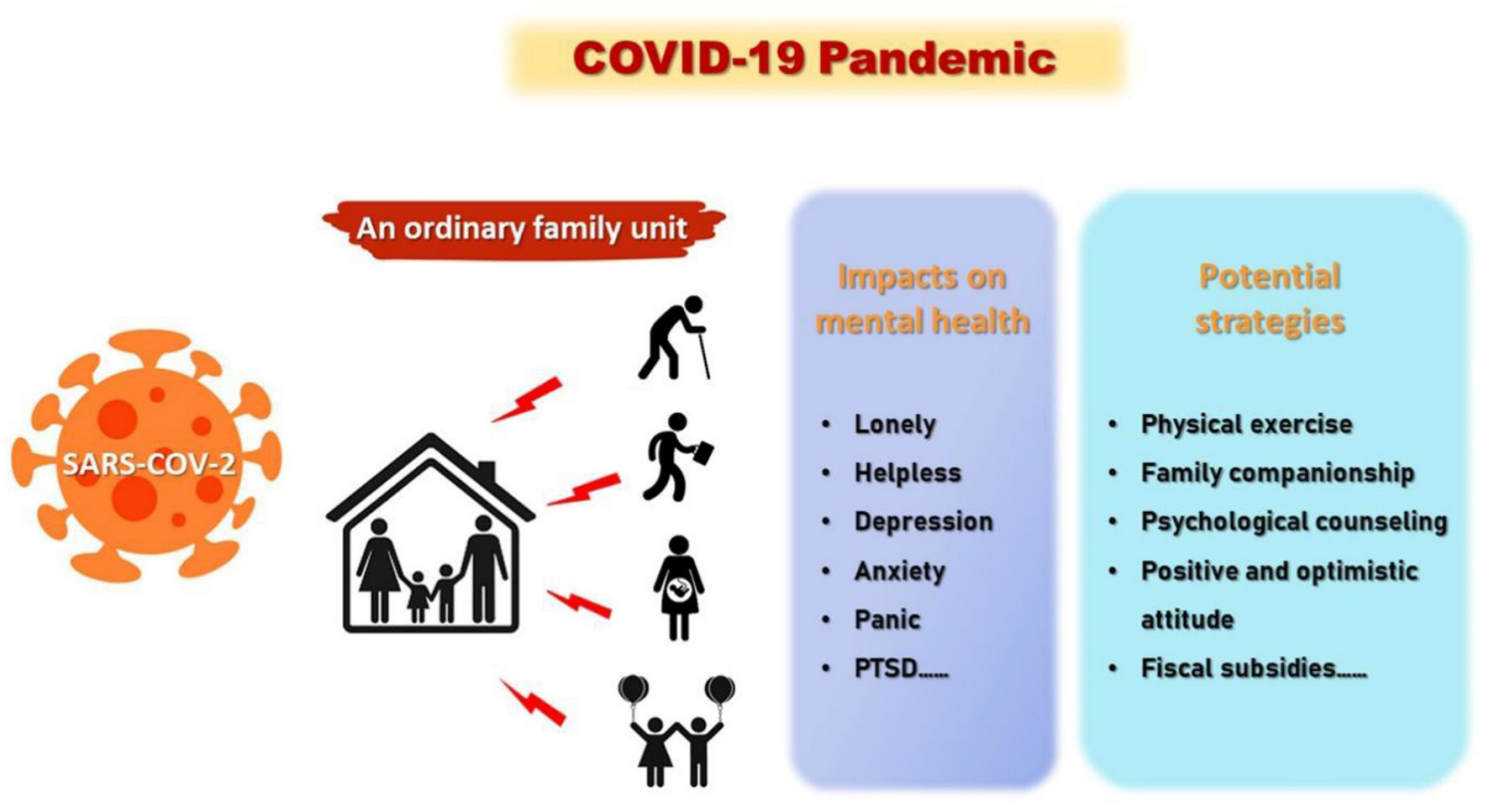
The effects of COVID-19 pandemic on mental health and corresponding solutions in different family members.

## Introduction

Coronavirus disease 2019 (COVID-19) was first reported in Wuhan City, China, in December 2019 (1). Owing to the exceptionally rapid transmission and robust infectiousness of the severe acute respiratory syndrome coronavirus 2 (SARS-CoV-2), and its high mortality and morbidity, the COVID-19 pandemic has swiftly led to a global public health crisis. As of November 3, 2021, there were ~248.007 million confirmed cases and over 5.0 million deaths in more than 200 countries worldwide (2).

COVID-19 has not only significantly affected the physical health of tens of millions of people worldwide but also affected individuals' mental health. Fear of the unknown virus, massive and long-term quarantine measures and economic losses, lack of basic supplies, cancelation of public events, and closing mass transit systems resulting from isolation have exacerbated stress and anxiety among the public, thus increasing individuals' risk of developing psychological disorders (3–6). According to the World Health Organization report on August 27, 2020, COVID-19 has affected the mental health of millions of people worldwide (7).

Since the outbreak of COVID-19, the mental health of medical workers and patients has been under intense scrutiny, and they have received a variety of psychological counseling and other treatments (8, 9). However, the mental health of the general population has received little attention concerning in comparison to healthcare workers and patients. To illustrate the public's psychological responses, we reviewed the potential causes and consequences of the adverse impacts of COVID-19 on the mental health of older adults, working adults, children and adolescents, and pregnant women by simulating an ordinary family unit, and put forward a series of targeted psychological crisis intervention measures. These addressed mental health issues would contribute to taking appropriate psychological interventions by the governments based on the characteristics of age-dependent groups. In fact, people of different ages experienced distinct psychological distress and emotional responses to the ongoing COVID-19 pandemic because they play different roles in a family.

## Older Adults

According to statistics from the Chinese Center for Disease Control and Prevention (China CDC), in the confirmed COVID-19 cases, the risk of prevalence and death rates increase with advancing age, i.e., the prevalence rate of people over 50-years-old in China was 53.6%. More seriously, the death rate for people over 50-years-old was 93.7% among 1,023 deaths (10).

Older adults are relatively psychologically fragile and vulnerable to the influence of the external environment, while the high morbidity rate and high mortality rate of the pandemic among the elderly further increase their psychological stress (11). First, over the past century, the sharp growth of aging populations has resulted in more aged people living alone. Aged people are more prone to have lonely and helpless feelings owing to a lack of emotional support from their children. Such feelings could be exacerbated and even result in mental disorders during the COVID-19 pandemic (11). Second, aging has been a major risk factor for chronic diseases, including cardiovascular diseases, diabetes, cancer, and neurodegenerative diseases (12, 13). These common morbidities render aged people more susceptible to the SARS-CoV-2 virus. However, owing to the isolation at home during the pandemic and the inability to go to the hospital for treatment, many older adults with chronic age-related diseases are more worried about their physical health, which further aggravates the prevalence of mental illness (12, 14). Third, as mandatory quarantine measures are taken, older adults may show a series of psychological disorders when changing their daily routines, such as reducing outdoor activities and social interactions during the COVID-19 pandemic (3, 11). Finally, containing the spread of the virus has entailed a dramatic shift from face-to-face to remote consulting for mental health professionals (11). Unfortunately, few older people are proficient in using internet services, which poses great challenges to their ability to access mental health services.

To lessen the potential impacts of psychological health and mental stress associated with the ongoing COVID-19 pandemic on aged people, several measures should be considered by family members and local governments. First, older adults could live together with their families to facilitate timely emotional communication and reduce loneliness. Alternatively, elders can also stay connected to friends and family members via traditional means (wired phones and letters) or advanced means (email, WeChat, online voice, and video chat) of communication (11, 15). Second, through community-based integrated care approaches, regular medical care and protective measures for those with chronic age-related diseases should be provided by community-based resources such as social services. Furthermore, timely and effective communication and targeted psychological interventions among multidisciplinary mental health teams about COVID-19 should be provided for elderly patients with persistent psychological symptoms. Third, occupational health experts can encourage the elderly to exercise to release their worries and anxiety and improve their immunity and cognitive performance (16). Elders should also be encouraged to obtain information from multiple regular media resources, such as television, newspapers, and radio, to relieve their anxieties and worries (17).

## Working Adults

In most ordinary families, the mental health of working adults can be easily overlooked during the pandemic. However, when facing unprecedented uncertainties, they may suffer great psychological pressure. First, the COVID-19 outbreak triggered a global economic recession, causing many unemployed or underemployed (18–20). Second, due to the stay-at-home policy during the pandemic in many countries, it is estimated that approximately one-half of the companies had more than 80% of their employees working from home during the early stages of the COVID-19 pandemic (20). Against this backdrop, both the limitations of the workspace and lack of positive social interaction are likely to have side effects that further aggravate people's stress (3). In China, an increasing number of adults are facing severe financial pressures, and some adults even cannot pay for basic requirements, including housing, food, and healthcare (21, 22). Such direct threats to the livelihood of working adults may harm their mental health more so than the ongoing disease itself. Third, a surge in domestic violence has been reported amidst the COVID-19 pandemic. Especially for females, employment and income source act as a buffer against violence; unemployment takes off this buffer and makes them vulnerable to violence at the hands of spouses (23). According to statistics from the National Commission for Women, India, there has been a 100 % increase in complaints related to violence against women after the nationwide lockdown was imposed in just 1 month (24). Finally, a return-to-work policy in many countries has been employed to compensate for the economic loss caused by the outbreak. However, the proportion of confirmed infections in many working adults has increased remarkably (25); therefore, for working adults, returning to work may increase their risk of infection, which will exacerbate their psychological burdens. Economic losses, family burdens, and domestic violence may lead to stress, anxiety, and other mental illnesses.

Given that the threats of psychological health deterioration to working adults cannot be neglected when we are fighting against the COVID-19 pandemic, several measures need to be implemented. First, people's financial losses should be identified during the isolation period by relevant government departments, and relief supplies and financial subsidies must be provided on a timely basis. Furthermore, the government should create more job positions for those who are unemployed or underemployed as soon as possible. In addition, for those who have changed their working style, it is necessary to help them get used to the new way of working and improve their ability and quality in time. Working adults should return to work in an orderly and periodic manner with governmental permission, as long as the COVID-19 pandemic is effectively controlled. Third, community social workers can play an active role in helping people cope with family issues (17). Besides, community psychological interventions and support might have some effects in reducing depressive and anxiety symptoms in adults during these stressful events (17). Notably, social support was the most important protective factor against psychological sequelae of the COVID-19 pandemic in working adults. It has been proposed that high-quality social support from family members and friends may alleviate anxiety and worry and enhance psychological and social relationships (26–29).

## Children and Adolescents

Among children and adolescents, the confirmed COVID-19 cases and death cases are less prevalent (2.1% of 44,672 confirmed cases) and less lethal (0.1% of 1,023 deaths) in China (10). Similar findings have been reported in other countries with more severe outbreaks (30). However, although the prevalence of COVID-19 among children is low, the extensive impact of the pandemic on children's mental health cannot be ignored. First, the periods of children and adolescents face a high degree of vulnerability to adverse environmental conditions (31), and the relative immaturity of the brain may make it particularly sensitive to stress-induced dysfunctions, with both immediate and lasting consequences on mental health (32). Second, to prevent the further spread of COVID-19, many countries have ordered school closures as an emergency solution (3, 4). Prolonged school closure can disrupt the normal social activities of young people with their classmates and teachers, which can have serious negative effects on their mental health, such as causing social phobia, anxiety, restlessness, and autism spectrum disorder (3, 33). Third, with many classes switched from offline to online, young people are spending significantly more time online, increasing their dependency on the internet (34). Additionally, internet engagement has increased among students because face-to-face interaction and activities are restricted. Students are more likely to be engaging in other online activities such as social media use and online gaming, which may be associated with internet addiction (35, 36). All these changes also exacerbate the conflicts between parents and children, which are associated with an increased risk of stress-related mental illness (37). In addition, children's ability to access too much information about the pandemic (infodemic) over the internet could easily cause panic (15).

Thus, close attention to children and adolescents is required to address these emergency issues effectively and avoid any long-term negative consequences for the rest of their lives. First, some low-risk areas began a phased resumption of classes with the outbreak under the initial control, which allowed children to play with their friends. Second, parents' supervision and guidance should be strengthened to prevent children from excessive internet use. Significantly, parents need to communicate more with their children to avoid further intensification of conflicts. Finally, to reduce children's excessive access to pandemic information by controlling their online time, parents should also keep a positive and optimistic attitude to avoid negative emotions affecting their children. Additionally, it is important to consider post-pandemic surveillance of mental disorders among children and adolescents (38). Furthermore, children and adolescents should stay physically active and engage in regular exercise to avoid the risk of physical and mental ill-health (38–42).

## Pregnant Women

Relevant studies indicated that pregnant women may be more susceptible to COVID-19 (43, 44). As special members of the families, therefore, we should also pay close attention to the psychological status of pregnant women during the pandemic. First, gestation is a time of significant psychological and physiological vulnerabilities; the higher psychosocial stress during this period can increase susceptibility to several mental disorders, including schizophrenia, mood disorders, high levels of anxiety, and autism (45). Second, during an outbreak, owing to the high potential risk of exposure to SARS-COV-2 in the hospital environment and the strict quarantine policy, many pregnant women were unable to conduct routine antenatal examinations or delivery, which may make them feel highly stressed and anxious (45, 46). Furthermore, some pregnant women worry that their babies will be infected after they are born during a pandemic. These fears have further deepened the postpartum anxiety of pregnant women (43, 44). Most noteworthy, the higher maternal psychosocial stress during gestation can directly affect the development of the fetus (47). A series of studies worldwide have reported a significant rise in the proportion of preterm (43) and stillbirth (48, 49) in pregnant women since the COVID-19 pandemic started. In addition, COVID-19 can also affect the health and well-being of mothers and their newborns by altering immune responses at the maternal-fetal interface (43).

In sum, owing to women being fragile physical condition during pregnancy, isolation at home for a long time, and worrying about one's baby, an increase in postpartum depression and other psychological problems is likely (46). Therefore, effective measures should be adopted to relieve the stress and anxiety of pregnant women. First, when the pandemic hit, some face-to-face consultations were substituted with online remote appointments to protect pregnant women from the coronavirus. However, medical workers cannot directly measure some critical physiological indicators of pregnant women, such as blood pressure, babies' heartbeat, and development status. Therefore, home antenatal examinations and delivery services should be provided by a specialized medical team to reduce visits to high-risk areas such as hospitals (46, 50). Second, during pregnancy, family companionship and psychological counseling are necessary, which can effectively reduce the psychological pressure on pregnant women. Moreover, to protect newborns, a mother should limit the number of people they have contact with (44). Finally, other online strategies, such as online mental health services or telemedicine, can help alleviate the psychological problems of pregnant women by communicating with others remotely (51–53).

## Conclusion

**Figure 1:**
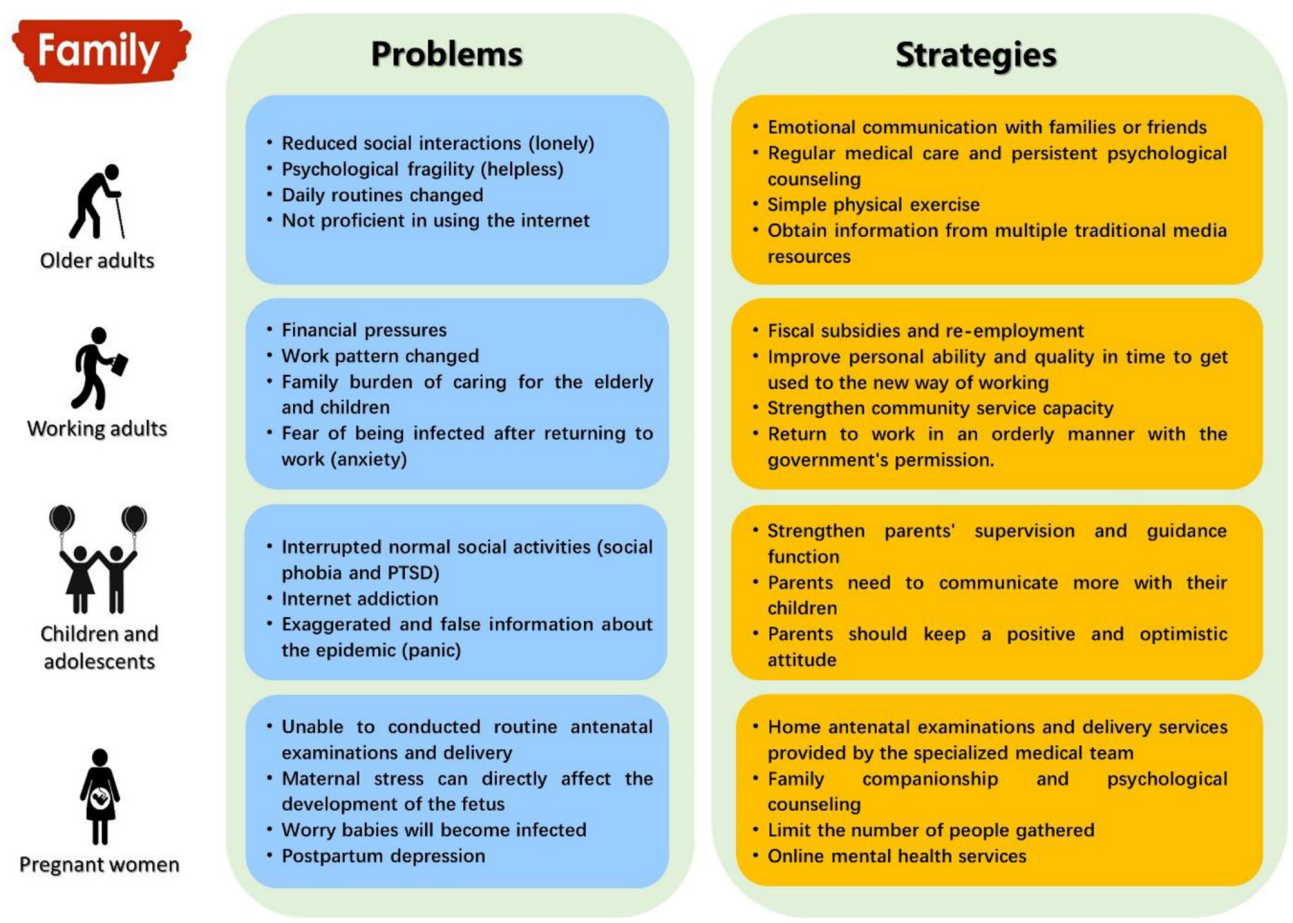
Impacts of the COVID-19 pandemic on individuals' mental health and corresponding strategies for mitigating these adverse effects among a stimulated ordinary family unit.

Among the general public, the sudden outbreak and prolonged duration of the COVID-19 pandemic have led to a series of adverse mental health problems. Given that there was limited information regarding mental health issues related to infectious diseases in past epidemics and pandemics, the general population received little attention concerning the diverse psychological impacts and mental health disorders. We analyzed the potential causes and consequences of the pandemic on mental health in different populations in an ordinary family unit with older adults, working adults, children and adolescents, and pregnant women, and then addressed several specific solutions to lessen the mental stress caused by the COVID-19 pandemic for each group (Figure 1). This information would contribute to establishing universal protocols and guidelines for the future, and inform appropriate and feasible guidance for successfully preventing the ongoing pandemic-related mental health problems to minimize the adverse impacts of the COVID-19 pandemic on mental health.

## Author Contributions

LW and GN: investigation and writing—original draft preparation. LZ: investigation. DL: writing—review and editing. DL and YW: conceptualization, supervision, and funding acquisition. All authors contributed to the article and approved the submitted version.

## Funding

This work was supported by the National Natural Science Foundation of China (NSFC, Grant No. 31770445) to YW, and NSFC (Grant No. 31672292) and the Natural Science Foundation of Hebei Province (NSFHB, Grant No. C2020205038) to DL, and NSFHB (Grant No. C2020205005) and China Post doctoral Science Foundation (Grant No. 2020M670685) and the Postdoctoral Research Program of Hebei Normal University to LW.

## Conflict of Interest

The authors declare that the research was conducted in the absence of any commercial or financial relationships that could be construed as a potential conflict of interest.

## Publisher's Note

All claims expressed in this article are solely those of the authors and do not necessarily represent those of their affiliated organizations, or those of the publisher, the editors and the reviewers. Any product that may be evaluated in this article, or claim that may be made by its manufacturer, is not guaranteed or endorsed by the publisher.
